# Familial hypercholesterolemia misinterpreted as juvenile idiopathic arthritis

**DOI:** 10.1186/1546-0096-9-S1-P228

**Published:** 2011-09-14

**Authors:** AJ Schou, P Toftedal, AE Christensen

**Affiliations:** 1 Hans Christian Andersen Children Hospital, Odense, Denmark

## Background

Early diagnosis is important in children with JIA to ensure early treatment and disease control. However, a wide range of diseases may mimic JIA causing overtreatment and delaying treatment of the underlying condition.

Familial hypercholesterolemia (FH) is an autosomal dominant genetic disorder with elevated low density lipoprotein (LDL) and total cholesterol due to mutations with loss of function of the LDL-receptor. The condition predisposes to early atherosclerosis, coronary disease and stroke. Severely affected individuals may present with tendinous xanthomata, which may be mistaken for rheumatic nodules.

## Aim

To describe a case of FH misinterpreted as JIA

## Methods

A ten-year-old girl was transferred to our department for further follow-up and treatment. Three years earlier she developed tumours in several tendons including both Achilles-tendons and arthralgia. The tumours were interpreted as rheumatic nodules, which was supported by histological examination. She was thus diagnosed with JIA and treated with MTX and NSAID with no effect, on the contrary the tumours continued enlarging. Upon transferral to our department very high LDL and total cholesterol levels were found. The diagnosis FH was confirmed genetically; the biopsies were re-evaluated and now described as chronic granulomatous inflammation surrounding cholesterol-deposits. Cholesterol-lowering treatment (simvastatin) was started lowering her blood values and the tumours were slowly regressing.

Her mother had normal cholesterol-levels, her father died young of unrelated causes.

## Conclusion

FH is yet another rare, but important differential diagnosis to JIA. The case illustrates how important it is to continuously pay attention and ensure that working diagnosis is correct.

